# Stem cell and niche regulation in human short bowel syndrome

**DOI:** 10.1172/jci.insight.137905

**Published:** 2020-12-03

**Authors:** Vered A. Gazit, Elzbieta A. Swietlicki, Miranda U. Liang, Adam Surti, Raechel McDaniel, Mackenzie Geisman, David M. Alvarado, Matthew A. Ciorba, Grant Bochicchio, Obeid Ilahi, John Kirby, William J. Symons, Nicholas O. Davidson, Marc S. Levin, Deborah C. Rubin

**Affiliations:** 1 Division of Gastroenterology, John T. Milliken Department of Medicine,; 2 Department of Surgery, and; 3 Department of Developmental Biology, Washington University School of Medicine, St. Louis, Missouri, USA.; 4 Veterans Affairs Medical Center, St. Louis, Missouri, USA.

**Keywords:** Gastroenterology, Adult stem cells

## Abstract

Loss of functional small bowel surface area following surgical resection for disorders such as Crohn’s disease, intestinal ischemic injury, radiation enteritis, and in children, necrotizing enterocolitis, atresia, and gastroschisis, may result in short bowel syndrome, with attendant high morbidity, mortality, and health care costs in the United States. Following resection, the remaining small bowel epithelium mounts an adaptive response, resulting in increased crypt cell proliferation, increased villus height, increased crypt depth, and enhanced nutrient and electrolyte absorption. Although these morphologic and functional changes are well described in animal models, the adaptive response in humans is less well understood. Clinically the response is unpredictable and often inadequate. Here we address the hypotheses that human intestinal stem cell populations are expanded and that the stem cell niche is regulated following massive gut resection in short bowel syndrome (SBS). We use intestinal enteroid cultures from patients with SBS to show that the magnitude and phenotype of the adaptive stem cell response are both regulated by stromal niche cells, including intestinal subepithelial myofibroblasts, which are activated by intestinal resection to enhance epithelial stem and proliferative cell responses. Our data suggest that myofibroblast regulation of bone morphogenetic protein signaling pathways plays a role in the gut adaptive response after resection.

## Abstract 2

LGR5+ stem cells are expanded and BMP signaling regulates the stem cell niche in human short bowel syndrome.

## Graphical abstract

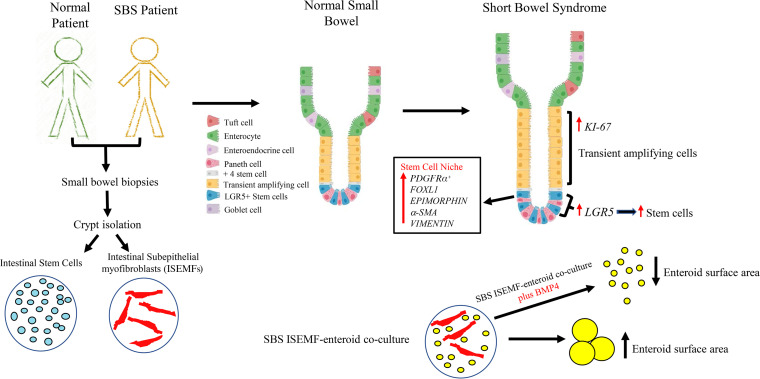

## Introduction

Loss of functional small bowel surface area following surgical resection for Crohn’s disease, ischemic injury, radiation enteritis, trauma, or malignancy may result in short bowel syndrome (SBS), an important cause of morbidity, mortality, and health care costs in the United States (1). Necrotizing enterocolitis and congenital disorders such as atresia and gastroschisis are the major causes of SBS in children. Following resection, the remaining small bowel epithelium mounts an adaptive response, resulting in increased crypt cell proliferation, increased villus height, increased crypt depth, and enhanced nutrient and electrolyte absorption (2). The functional and morphometric adaptive response is best described in animal models. In humans, this response is influenced by remnant small bowel length, the presence or absence of a colon, and underlying disease activity, yet even accounting for these factors, the response may be unpredictable and inadequate (1, 3). Even with an accurate assessment of remnant small bowel length, it may require from 2 to 5 years to determine which patients will wean off parenteral (intravenous) nutrition (4).

There are few studies of gut adaptation in human SBS, and these are inconclusive because of limited numbers of patients (5, 6). Patients with SBS have been reported to have an increase (7, 8) or no change (9–11) in crypt cell proliferation rates and morphologic adaptation after resection (reviewed in ref. 6). These studies, which are clinically heterogeneous, include studies of infants (7), jejunoileal bypass patients (8), and colon adaptation (9). Only 2 studies have focused on the small bowel (10, 11). These report modest numbers of patients (11) with limited data on crypt-villus morphometrics (10) and no analysis of stem cell and crypt proliferative responses. Furthermore, treatments for SBS are for the most part nonspecific and are directed toward symptomatic diarrhea control. Only one therapy has been developed that is specific to SBS, teduglutide. This glucagon-like peptide 2 analog has multiple proadaptive effects, including increasing crypt cell proliferation, crypt depth, and villus height. Teduglutide is effective in reducing parenteral nutrition (PN) requirements in properly selected patients, of whom a subset may completely wean from PN. Yet others remain at least partially PN dependent, are ineligible for this medication, or develop side effects (12). Thus additional therapies are needed. Critical interactions between luminal and host signaling pathways that modulate the stem cell/proliferative and functional adaptive response have yet to be elucidated. Accordingly, understanding the signaling pathways that modulate intestinal stem cell interactions with the local (niche) environment following small bowel resection is a critical unmet need.

Mesenchymal cells play an important role in the gut stem cell niche, in health and in injury and disease (13, 14). We and others have shown that intestinal subepithelial myofibroblasts (ISEMFs) have stem cell niche activity in mouse gut and support stem cell survival and growth of cocultured epithelial stem cell–derived enteroids (15–17). Our previous studies suggest an important role for epimorphin, a syntaxin that regulates growth factor and cytokine secretion from ISEMFs (15–17). Also cell populations in mice marked by FOXL1 (18, 19), PDGFRα (20), Gremlin1 (21), GLI1 (22, 23), and CD34/GP38 (24) have significant niche activity and are required for stem cell proliferation. Single-cell RNA-Seq analysis of colon biopsies from patients with ulcerative colitis has revealed novel mesenchymal populations with stem cell niche activity that may serve as targets for therapy (13). However, the characterization and role of these stromal populations in human gut and in SBS are unknown.

Here we address the hypotheses that a subset of intestinal stem cells are expanded and that the stem cell niche is regulated following massive gut resection in human SBS. The magnitude and phenotype of the adaptive stem cell response is regulated by stromal niche cells, including ISEMFs that are activated by intestinal resection to enhance epithelial stem and proliferative cell responses. Our data suggest that ISEMF regulation of bone morphogenetic protein signaling pathways plays a role in the gut adaptive response following small bowel resection.

## Results

### Human SBS stem cell isolation.

To examine the effects of massive small bowel resection and resulting SBS on small intestinal stem cell populations and the stem cell niche in humans, we collected small bowel biopsies from 17 patients with SBS and from 24 control subjects, obtained as part of routine clinical care (see Methods). The demographics of the SBS population, including age, sex, race, etiology of SBS, and diet history (oral vs. parenteral nutrition) are summarized in Table 1, and details are reported in Supplemental Table 1; supplemental material available online with this article; https://doi.org/10.1172/jci.insight.137905DS1 SBS patients included those fed an oral diet only or fed partially or completely with PN for various times postresection. There were 9 patients with more than 50% colon remaining in continuity, 1 patient with a sigmoid colostomy who had less than 50% colon remaining, and 7 patients with small bowel ostomies. SBS resulted from Crohn’s disease (*n* = 6), radiation enteritis (*n* = 3), ischemia (*n* = 4), fistula (*n* = 1), trauma (*n* = 1), and adhesions (*n* = 2). There were 7 men and 10 women, with an average age of 59.6 years; 6 patients were fed with oral nutrition only.

### Transit amplifying cells and leucine rich repeat containing G protein-coupled receptor 5–positive intestinal stem cells are increased in patients with SBS.

Total RNA was isolated from small bowel biopsies, and expression of transit amplifying proliferative crypt cell gene markers (*KI67* and *CD44*) and stem cell gene markers (leucine rich repeat containing G protein-coupled receptor 5 [*LGR5*], SRY-box transcription factor 9 [*SOX9*], and BMI1 proto-oncogene, polycomb ring finger [*BMI1*]) was analyzed by quantitative reverse transcription PCR (qRT-PCR).

**Figure 1:**
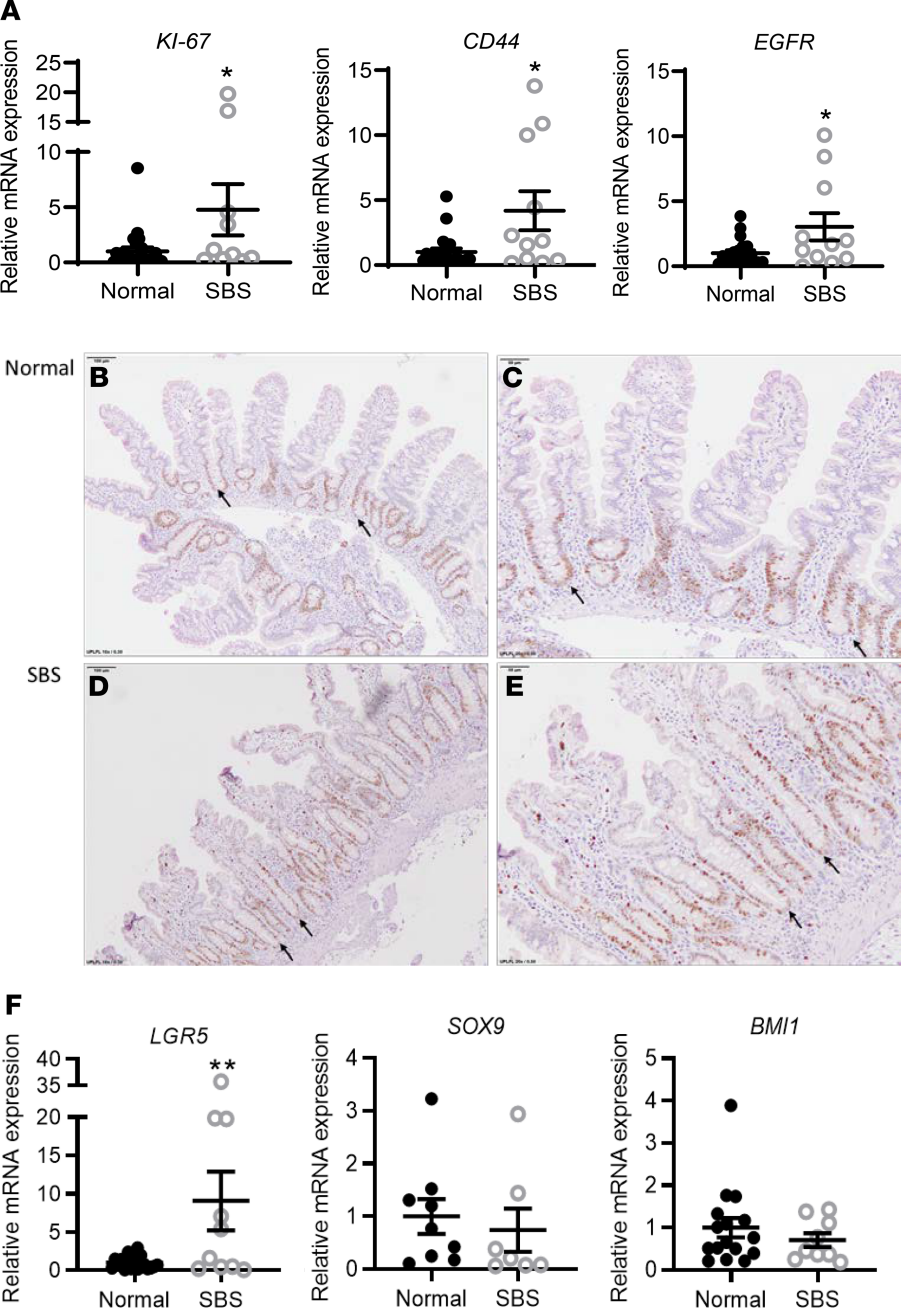
Increased crypt cell proliferation in small bowel biopsies from SBS patients compared with normal control subjects. (**A**) Increased *KI67* (*P = 0.027), *CD44* (*P = 0.012), and *EGFR* (*P = 0.011) mRNA levels in SBS versus normal small bowel, quantified by qRT-PCR. (**B**–**E**). Representative images of immunohistochemical analysis of KI67 expression (brown cells) in normal (**B** and **C**) and SBS (**D** and **E**) small bowel. Arrows depict representative full-length intestinal crypts. Scale bars: 100 μm (**B** and **D**), 50 μm (**C** and **E**). (**F**) Increased *LGR5* mRNA levels (**P = 0.007) in SBS versus control small bowel. The mRNA levels of +4 position stem cell markers *SOX9* and *BMI1* are unchanged. SBS: *n* = 9–12; normal: *n* = 16–24. Data are means ± SEM. Statistical analysis by Student’s *t* test.

Expression of *KI67* and *CD44* mRNA was significantly increased in SBS compared with normal patient biopsies (Figure 1A), consistent with increased crypt transit amplifying cell proliferation (a hallmark of mouse models of adaptation following resection; refs. 25, 26). EGFR/RAS/MAPK signaling is a major driver of proliferation that promotes the exit of stem cells into the transit amplifying cell population (27). Therefore we measured EGFR mRNA expression by qRT-PCR and observed a significant increase in EGFR expression in SBS (Figure 1A). To correlate mRNA with cell-specific expression, we analyzed KI67 expression by immunohistochemical analysis (Figure 1, B–E). SBS biopsies showed lengthening of the crypts compared with normal biopsies, and immunohistochemistry showed increased crypt expression in the transit amplifying population associated with increased crypt depth in patients with SBS (Figure 1, B–E).

To determine whether the increase in transit amplifying cell proliferation also reflected an expansion of stem cell populations, we examined expression of *LGR5*, the definitive marker of the rapidly dividing gut crypt base stem cell population that replenishes epithelial cells lost by daily cell turnover (Figure 1F). We observed a significant increase in *LGR5* expression in SBS biopsies compared with normal patients.

In contrast, expression of the quiescent +4 stem cell population marker, *SOX9* (28), was unchanged in SBS, and expression of *BMI1*, a putative marker of the quiescent +4 stem cell population and of enteroendocrine cells that dedifferentiate when injured to become stem cells (29), was not significantly increased (Figure 1F).

**Figure 2:**
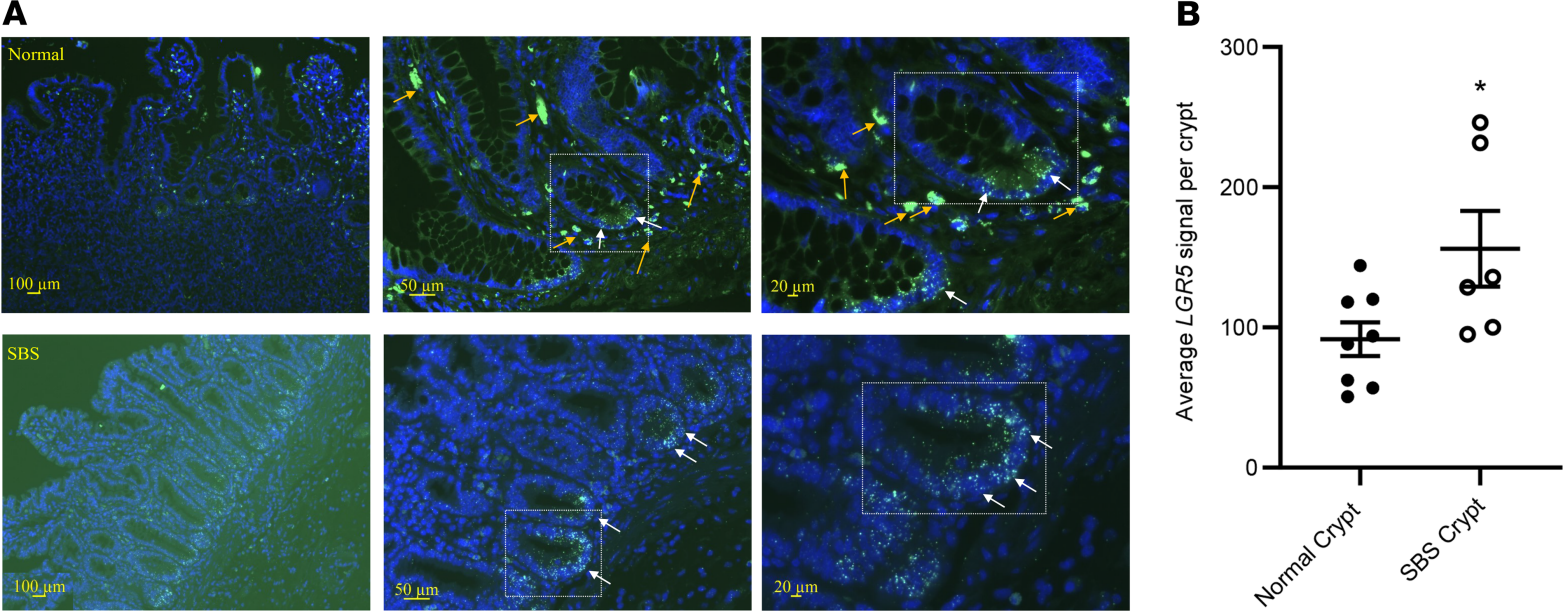
*LGR5* mRNA expression is increased in the crypt base of SBS compared with control ileum. (**A**) In situ hybridization by RNAscope to detect *LGR5* mRNA (small green dots, white arrows) on sections of normal ileum (top panels) and SBS ileum (bottom panels). Slides are counterstained with DAPI for nuclei (blue). Orange arrows denote lamina propria cells that have intrinsic autofluorescence leading to artifact. (**B**) Quantitation of the number of *LGR5*^+^ dots per crypt from sections of normal (*n* = 8) and SBS (*n* = 6) ileal biopsies (*P = 0.034). Data are means ±  SEM. Statistical analysis by Student’s *t* test.

We next examined cell-specific *LGR5* expression in SBS biopsies by in situ hybridization analysis using RNAscope fluorescence assays. As expected, *LGR5* mRNA was located at the base of the crypts (Figure 2A). Quantitation of cellular *LGR5* mRNA expression by ImageJ (NIH) analysis showed significantly increased expression in SBS (Figure 2B; *P* = 0.034).

### Increased stem cell activity in human SBS.

**Figure 3:**
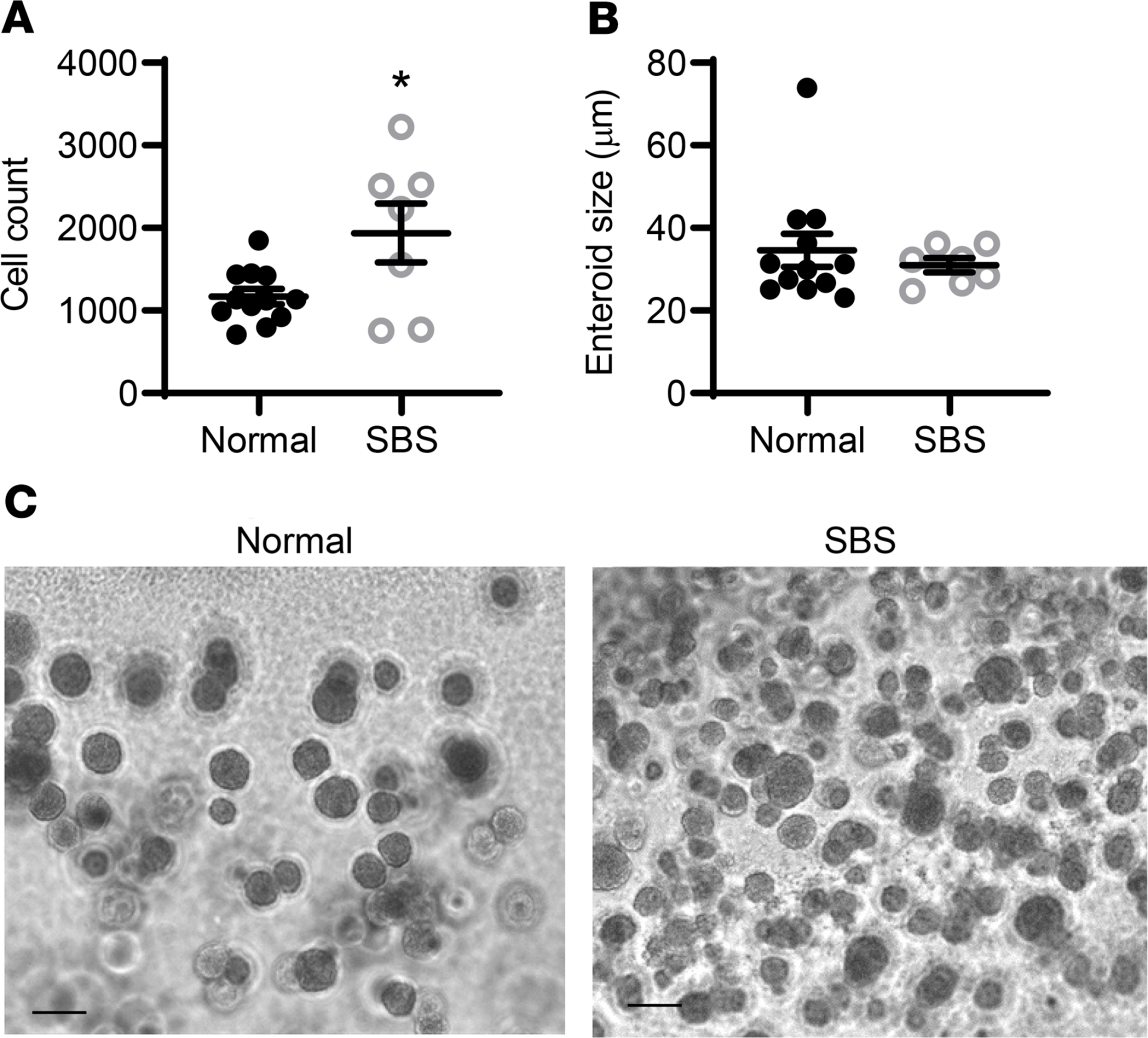
Stem cell expansion in small intestine from patients with SBS. Stem cells isolated from small bowel biopsies from patients with SBS and from normal controls were plated for stem cell enteroid initiation assays as per Methods. The number of enteroids per well and enteroid size (cross-sectional area) were quantified using the Cytation 3 plate reader. (**A**) Average number of stem cell–derived enteroids from biopsies from normal patients (*n* = 12) and SBS patients (*n* = 7) that were grown in Matrigel for 7 days. *P = 0.0178. Twenty wells were analyzed for each patient, and experiments were repeated at least twice. (**B**) Average cross-sectional area of SBS (*n* = 7) versus normal (*n* = 12) patient stem cell–derived enteroids. All data are means ± SEM. Statistical analysis by Student’s *t* test. (**C**) Photomicrographs of normal (left panel) and SBS (right panel) stem cell–derived enteroids. Scale bar: 50 μm, imaged by Zeiss Axiophot with Apotome 2 microscopy.

To determine whether the increase in LGR5 expression was reflected by a functional increase in stem cell activity, we performed stem cell–initiated enteroid formation assays and compared the ability of SBS compared with normal patient stem cells to form enteroids (Figure 3). Crypts were isolated from SBS and normal intestinal biopsies, dissociated, and cultured to produce stem cell–derived enteroids. These were passaged twice, released from Matrigel, and frozen. To perform stem cell enteroid initiation assays, stem cells from SBS (*n* = 7) and healthy patients (*n* = 12) were obtained from frozen stem cell stocks. Single stem cells were plated in Matrigel, and the number of surviving enteroids was imaged and counted on day 7 after plating.

Patients with SBS had a significant increase in the number of stem cell–derived enteroids compared with healthy subjects (Figure 3, A and C), providing a functional correlation for the observation that *LGR5* mRNA levels were increased in SBS (Figure 1 and Figure 2). These results also indicate that even after passaging and freezing, SBS stem cells retained the observed in vivo phenotype for expansion compared with normal stem cells. Enteroid size (cross-sectional area), which reflects growth of each surviving stem cell–derived colony, was unchanged in SBS compared with normal subjects (Figure 3B).

### WNT ligand and target gene expression in SBS.

**Figure 4:**
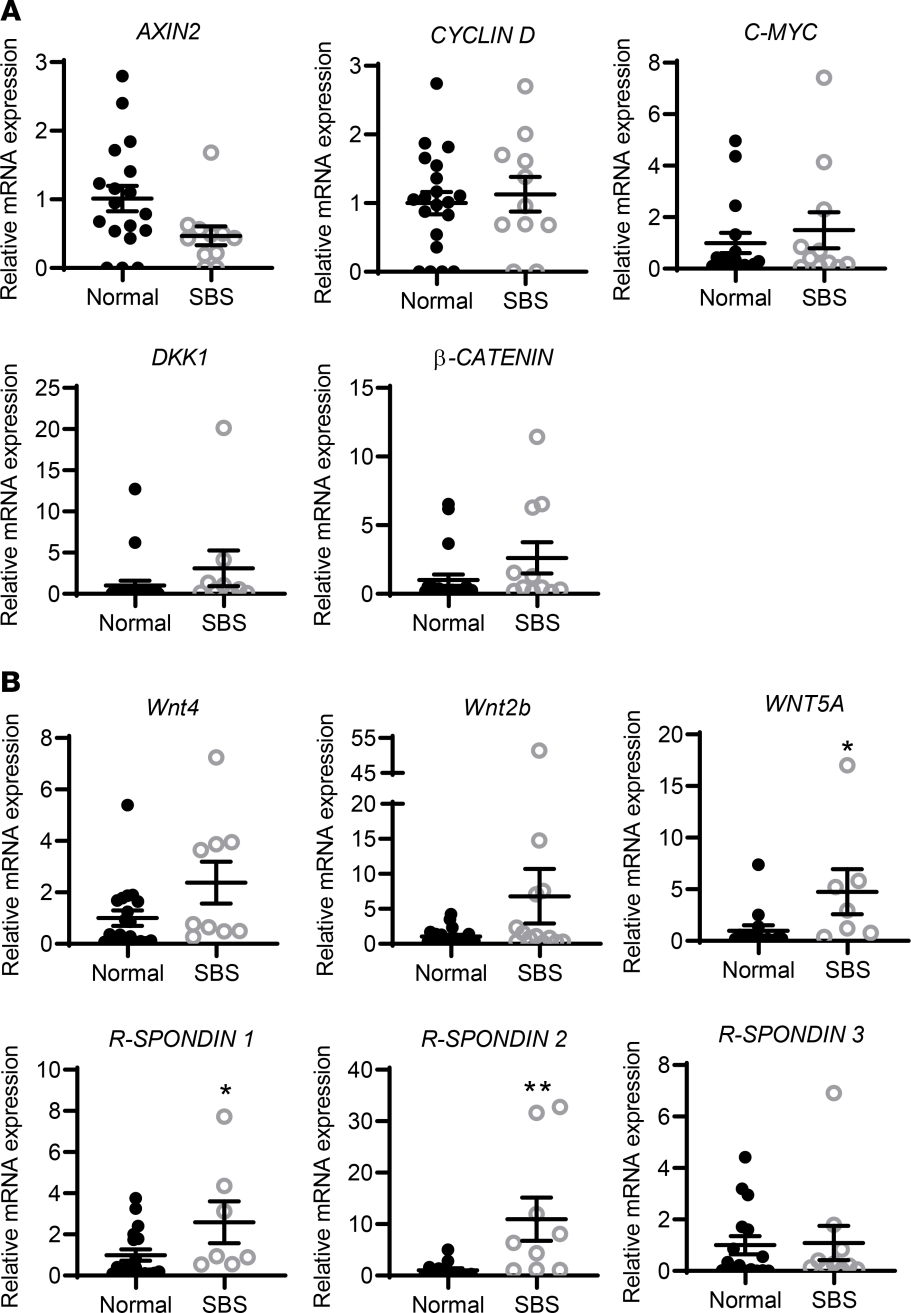
*WNT* signaling pathway component gene expression in SBS versus control small intestinal biopsies. Relative mRNA levels were measured by qRT-PCR. (**A**) *WNT* target gene analysis shows that *AXIN2* mRNA expression showed a trend to be decreased in SBS versus control ileum, *P* = 0.054, but other target gene mRNA levels are unchanged. (**B**) *WNT5A* and *R-SPONDIN1* and *2* mRNA levels are increased in SBS. Normal: *n* = 13–24; SBS: *n* = 7–12. *WNT5A* *P = 0.049; *R-SPONDIN1* *P = 0.048; *R-SPONDIN2* **P = 0.01. Data are means ± SEM. Statistical analysis is by Student’s *t* test.

LGR5^+^ stem cell proliferation and commitment to specific differentiation pathways are regulated by WNT, BMP, and NOTCH signaling (30), contributing to autocrine and niche regulation of stem cell maintenance and expansion. To begin to assess WNT signaling activity in SBS, we examined expression of downstream WNT target genes including *AXIN2*, *cyclin D1*, and *C-MYC*; WNT signaling pathway components including dickkopf WNT signaling pathway inhibitor 1 (*DKK1*) and *β-CATENIN*; and WNT agonists and ligands by qRT-PCR. Although LGR5^+^ cell populations were expanded, there was no change in *WNT* target gene expression, including *cyclin D1*, *C-MYC*, *DKK1*, or *β-CATENIN*, and *AXIN2* expression showed a trend to be decreased in SBS compared with normal biopsies (Figure 4A, P** = 0.054). In contrast, expression of LGR4–6 receptor ligands/WNT agonists *R-spondin1* and *R-spondin2* was significantly increased in SBS versus normal biopsies (Figure 4B). *R-spondin3* expression was unchanged. R-spondin1 is expressed in epithelial and mesenchymal cells; R-spondin2 and R-spondin3 are expressed predominantly in mesenchyme (31, 32). The mRNA expression of the mesenchymal *WNT* ligand *WNT5a* was also increased.

### BMP signaling pathways are regulated in human SBS.

Increased ligand expression without concomitant increased WNT signaling activity suggested that the SBS mesenchyme generates a stromal niche adaptive response following resection to facilitate stem cell expansion, but WNT signaling activity may be inhibited by other counterregulatory pathways such as the BMP pathway. BMP signaling plays a role in the small intestinal stem cell niche, regulating crypt cell proliferation and epithelial cell differentiation (30). BMP signaling activity is lowest at the base of the crypt, due to enriched mesenchymal expression of BMP inhibitors including noggin, gremlin, and chordin (33, 34), and increases along the crypt/villus axis as cells exit the crypt and differentiate during migration onto the villus, inhibiting stem and crypt cell proliferation and promoting epithelial cell differentiation.

**Figure 5:**
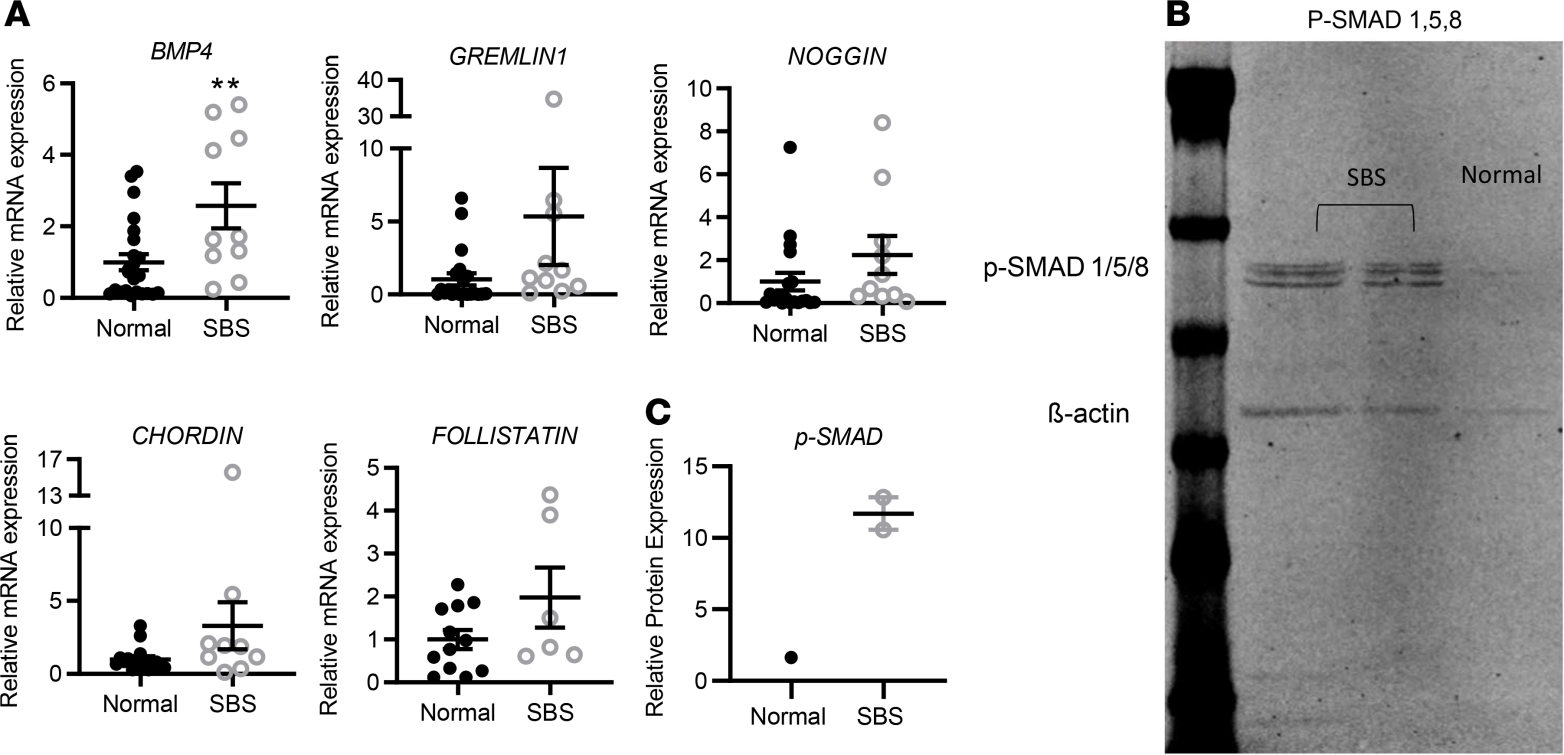
BMP signaling pathway mRNA expression in SBS versus normal small bowel biopsies. (**A**) Total RNA was isolated from normal (*n* = 12–23) and SBS (*n* = 6–10) small bowel biopsies, and mRNA levels were quantified by qRT-PCR. *BMP4* **P = 0.009; data are means ± SEM. Statistical analysis by Student’s *t* test. (**B**) Immunoblot to detect p-SMAD1,5,8 expression in SBS compared with normal ileum. First lane (left), molecular weight markers; second and third lanes, SBS; *n* = 2 patients pooled per lane); and fourth lane, normal (*n* = 2 patients pooled). (**C**) Quantitation of relative p-SMAD1,5,8 protein expression was performed by optical densitometric (LI-COR) analysis of p-SMAD1,5,8 and β-actin bands. p-SMAD1,5,8 expression was normalized to β-actin, and SBS p-SMAD1,5,8 expression was calculated relative to normal ileal expression.

We quantified mRNA expression of *BMP4*, *BMP2*, and *BMP7* and *BMP* inhibitors *noggin*, *gremlin1*, *chordin*, and *follistatin*. Expression of *BMP4* mRNA, which inhibits stem and crypt cell proliferation and promotes epithelial differentiation, was significantly increased in SBS intestine (Figure 5A). *BMP2* and *BMP7* showed no change (data not shown). Conversely expression of BMP inhibitors *gremlin1*, *noggin*, *chordin*, and *follistatin* were unchanged in SBS versus controls.

To further examine whether BMP signaling activity is regulated in SBS, we next examined expression of phosphorylated SMAD1,5,8 (p-SMAD1,5,8), the major downstream target of BMP signaling by immunoblot. We found that SBS biopsies had increased p-SMAD1,5,8 expression compared with normal gut, which showed low basal levels of p-SMAD1,5,8 expression (Figure 5B, Supplemental Figure 1). Thus BMP signaling activity appears to be increased overall in SBS mucosa.

### Mesenchymal stem cell niche populations in SBS.

To further explore the cellular basis for the increase in mesenchymal WNT ligand, WNT agonist (R-spondins), and BMP4 expression, we examined the adaptive SBS response of gut mesenchymal (stromal) cell populations that have stem cell niche activity (13, 18–20, 22, 24, 27) by cell marker expression analyses using qRT-PCR. Gut mesenchymal populations include ISEMFs, which are smooth muscle α-ACTIN–positive (SMA-positive), vimentin-positive, and desmin-negative cells that express epimorphin, a myofibroblast protein that regulates growth factor secretion (15, 16); and FOXL1^+^ telocytes, which also have been shown to play a role in the stem cell niche in mouse models and in human inflammatory bowel disease (13). Also CD34^+^/GP38^+^ cells (24) and SMA^–^/ PDGFRα^+^/R-spondin3^+^ cells have been described in mouse intestine (20). These cells all express PDGFRα (27) and are the source of WNT ligands and R-spondins, including WNT2b, WNT4, and WNT5a; R-spondin1, 2, and 3; and BMPs (27).

**Figure 6:**
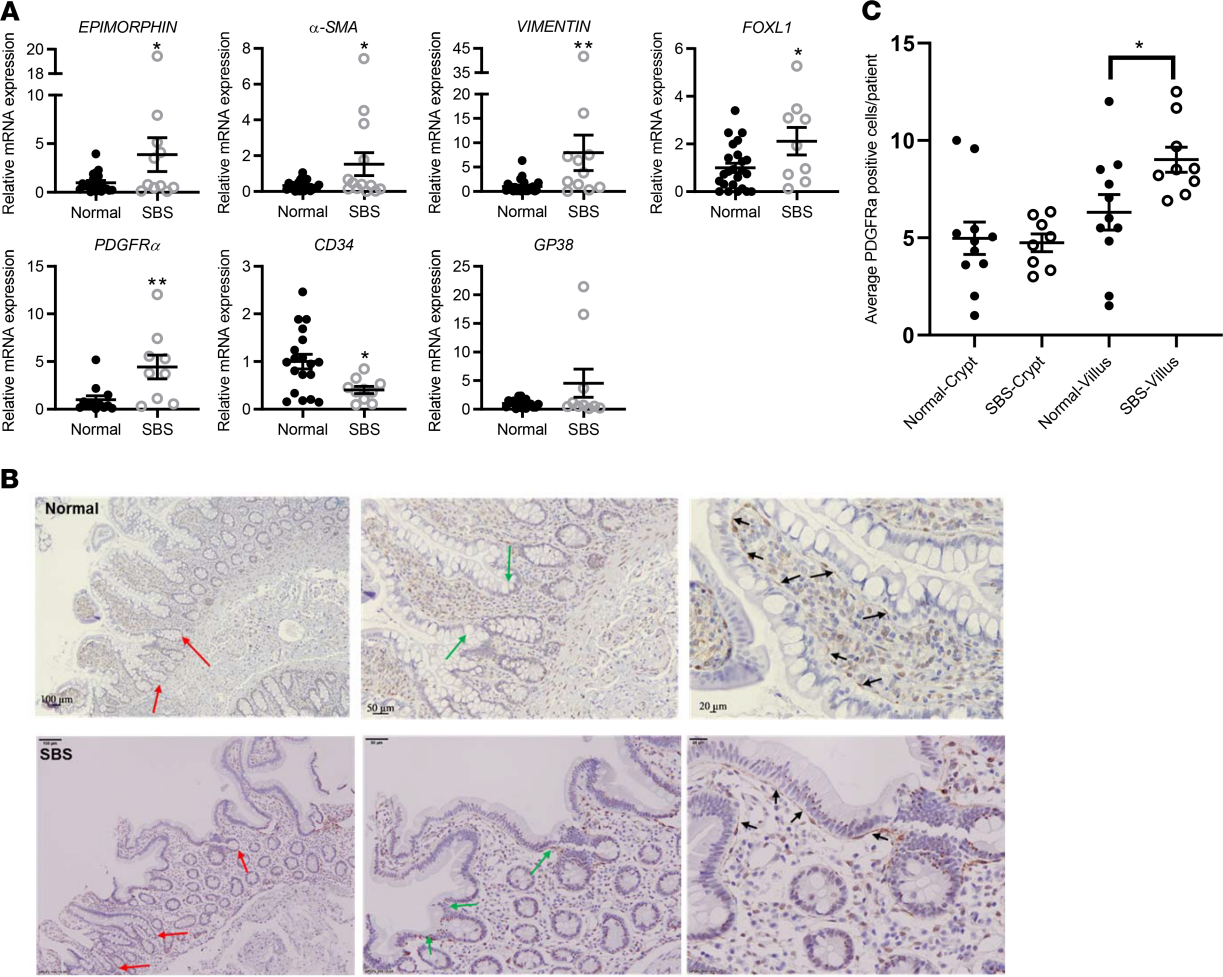
Mesenchymal cell marker gene expression analyses. Stem cell niche mesenchymal cell marker gene expression by qRT-PCR analysis of mRNA from normal (*n* = 12–24) and SBS (*n* = 9–13) small bowel biopsies. (**A**) A subset of mesenchymal markers including myofibroblast markers (*epimorphin*, *αSMA*, *vimentin*) and *PDGFRα* and *FOXL1* exhibited increased mRNA levels in SBS versus normal small bowel. *Epimorphin* *P = 0.042; *αSMA* *P = 0.024; *vimentin* **P = 0.012; *FOXL1* *P = 0.049; *PDGFRα* *P = 0.009; *CD34* *P = 0.025. Data are means ± SEM. Statistical analysis by Student’s *t* test. (**B**) PDGFRα^+^ cells (brown stained cells) in normal (upper panel) and SBS (lower panel) ileal biopsies detected by immunohistochemical analysis, using a mouse anti-human PDGFRα antibody. Black arrows on high-power views (right panels) show PDGFRα^+^ cells subjacent to the villi. Red arrows on low-power view (left panels) depict the crypts. Green arrows (middle panels) show the crypt-villus junction. Scale bars: 100 μm (left), 50 μm (middle), 20 μm (right). (**C**) The number of crypt- and villus-associated PDGFRα^+^ cells was quantified in SBS patient crypts (*n* = 8) and villi (*n* = 9) and normal patient crypts (*n* = 11) and villi (*n* = 11). Each data point represents the average percentage of positive cells per patient. Only intact villi and crypts on each histologic section were analyzed. *P = 0.031. Data are means ± SEM. Statistical analysis by Student’s *t* test.

Expression of *vimentin*, *SMA*, and *epimorphin* mRNA was significantly increased in SBS biopsies by qRT-PCR (Figure 6A) consistent with expansion and/or activation of myofibroblasts, which express mesenchymal WNTs and R-spondin1, 2, and 3. In addition, *FOXL1* and *PDGFRα* mRNA expression was increased in SBS versus control small bowel (Figure 6); in contrast, *CD34* expression was reduced and expression of *GP38* and *Rspondin-3* (as per Figure 4B) was unchanged. Therefore, subpopulations of some, but not all, PDGFRα^+^ cells respond to loss of functional small bowel surface area, suggesting cell-specific adaptation.

To examine the cellular basis for the increased expression of multiple mesenchymal cell markers, we performed immunohistochemical analysis to detect PDGFRα^+^ cells. We noted a significant increase in PDGFRα^+^ cells in the villus cores of SBS compared with control biopsies (Figure 6, B and C).

### Outlier and subgroup analyses.

Outlier analyses followed by additional subgroup analyses were performed for all the data reported in Figure 1, Figure 4, Figure 5, and Figure 6 to determine whether outliers have a unique clinical phenotype, as per Methods. We found no association of outliers with the presence or absence of a colon in continuity, age, sex, intestinal location (duodenum, jejunum, or ileum), or oral feeding versus PN.

### SBS ISEMFs increase growth of cocultured SBS enteroids.

**Figure 7:**
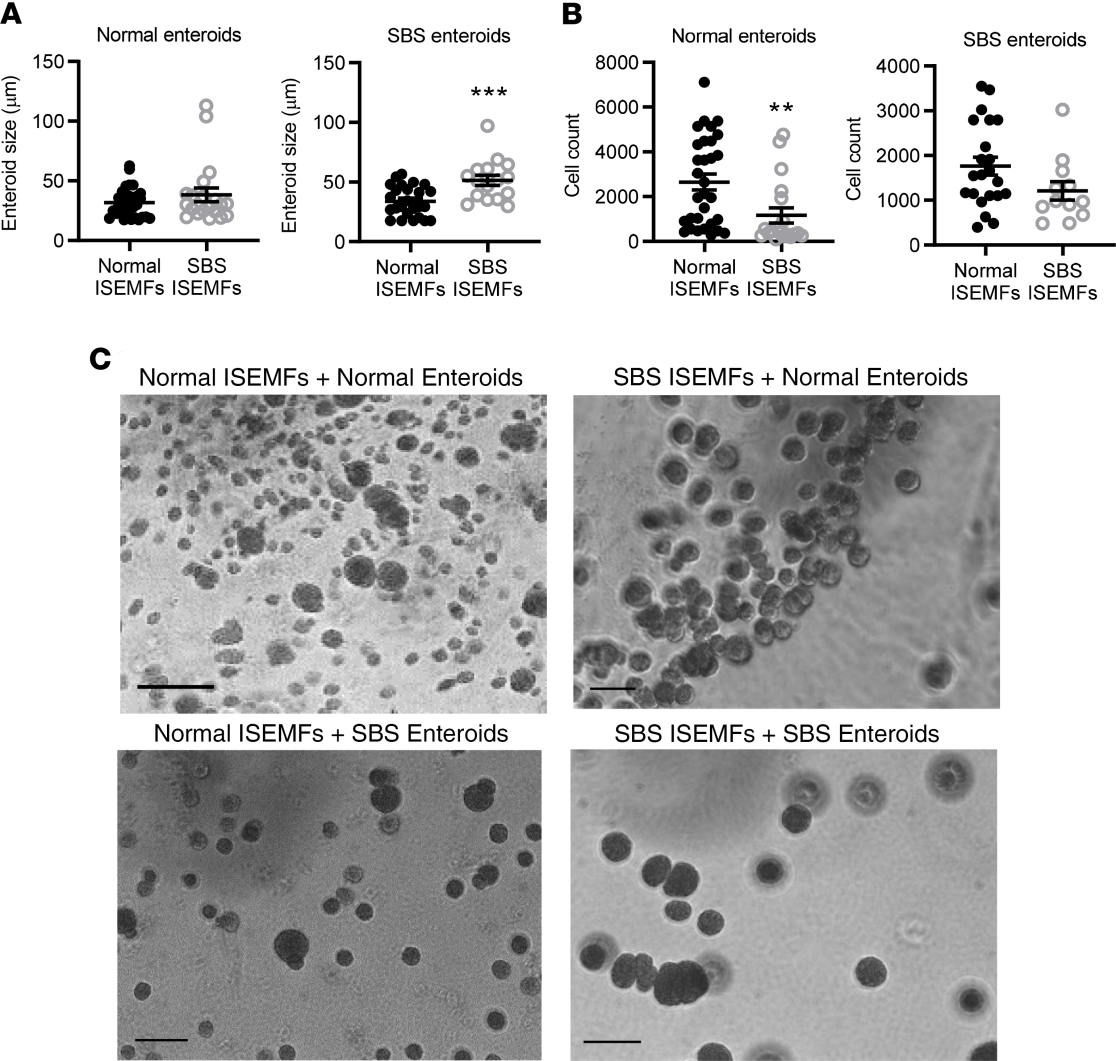
SBS ISEMFs increase surface area of cocultured ileal SBS enteroids. Normal enteroids (*n* = 9 patients) and SBS enteroids (*n* = 6 patients) were cocultured for 7 days with normal (*n* = 4 subjects) or SBS (*n* = 4 patients) ISEMFs. Enteroid cross-sectional area (**A**) and the number of enteroids (cell count, **B**) were quantified by Cytation 3. (**A**) SBS enteroid size ***P = 0.0006; (**B**) Normal enteroid cell count **P = 0.0068; SBS enteroids = cell count, NS. Data are means ± SEM. Statistical analysis by Student’s *t* test. (**C**) Images of ISEMF-enteroid cocultures by Zeiss Axiophot microscope with Apotome 2 attachment. Scale bar: 50 μm.

To further explore mesenchymal cell–specific effects on stem cell proliferation and the stem cell niche, we isolated human ISEMFs from SBS and normal control patient biopsies. ISEMFs were chosen for further study because these cells expressed activation markers in SBS compared with normal intestine, (*α-SMA*; Figure 6). ISEMFs were cocultured with SBS and normal stem cells, and the number and size (surface area) of surviving enteroids were measured after 7 days in coculture (Figure 7). We found that SBS enteroid size increased when cocultured with SBS ISEMFs compared with cocultures with normal patient ISEMFs (Figure 7, A and C). In contrast, there was no effect on the size of normal enteroids when cocultured with SBS compared with normal ISEMFs, suggesting a specific interaction between SBS ISEMFs with SBS enteroids.

The number of stem cell–derived enteroids was significantly lower in normal enteroid–SBS ISEMF cocultures compared with normal enteroid–normal ISEMF cocultures (Figure 7B). In contrast, there was no change in SBS enteroid cell count when cocultured with normal versus SBS ISEMFs, again suggesting that SBS enteroids have cell-autonomous changes that alter their response to coculture with ISEMFs.

**Figure 8:**
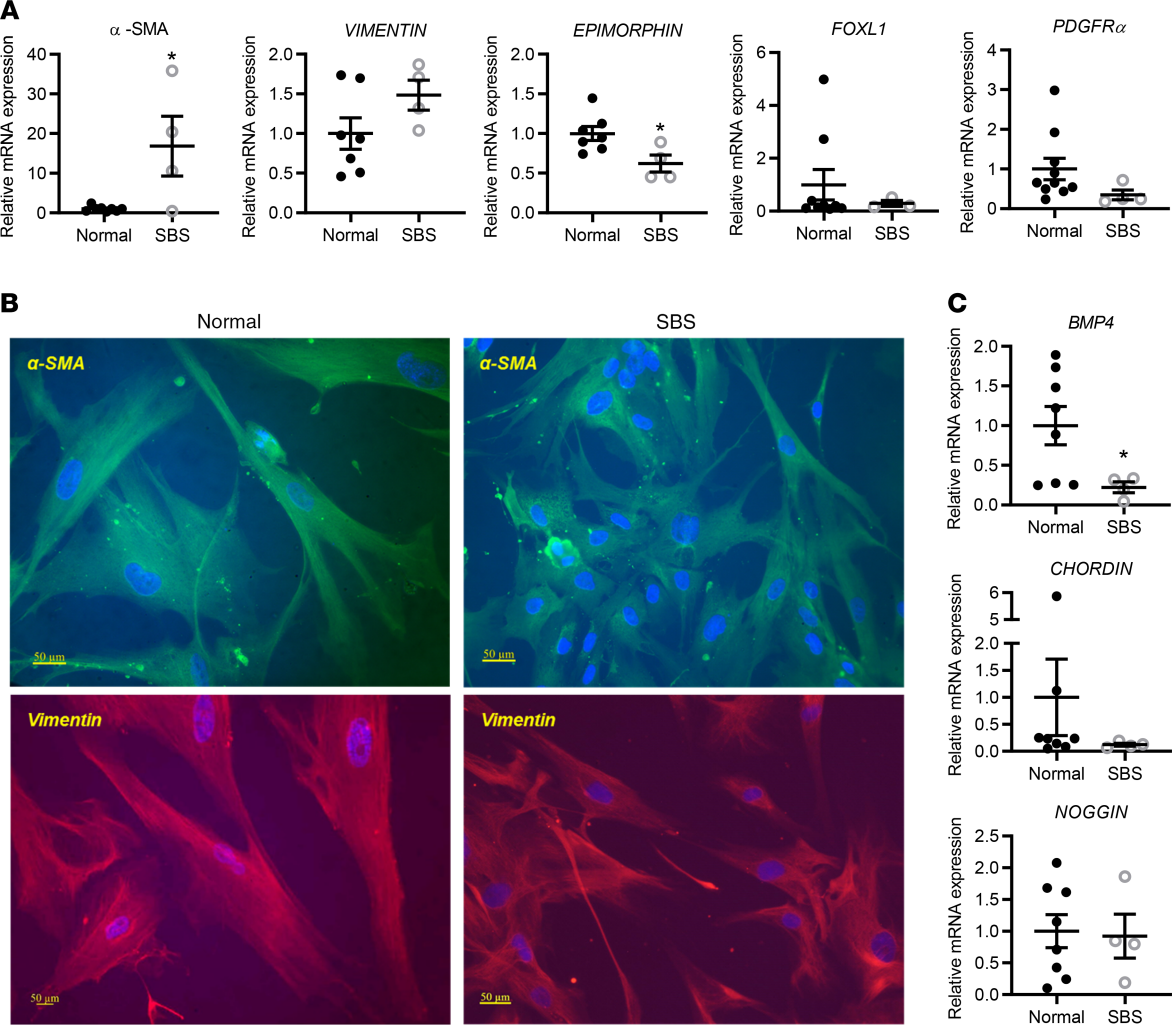
Mesenchymal marker and BMP signaling pathway gene expression in normal and SBS ISEMFs. (**A**) SBS ISEMFs have increased *α-SMA* (*P = 0.0109) but reduced *epimorphin* mRNA expression (*P = 0.0264) compared with normal ISEMFs. Data are mean ± SEM. Statistical analysis by Student’s *t* test. There was no change in *FOXL1* or *PDGFRα* mRNA expression. (**B**) Immunohistochemical analysis of human normal and SBS ISEMFs shows expression of myofibroblast marker genes *α-SMA* and *vimentin*. (**C**) BMP4 signaling pathway gene expression in SBS ISEMFs. ISEMFs from normal and SBS patient biopsies were isolated as per Methods. *BMP4* mRNA is reduced in SBS. *P = 0.0528. SBS, *n* = 4; normal, *n* = 7–8. Data are means ± SEM. Statistical analysis by Student’s *t* test.

To elucidate the mechanisms underlying the effect of SBS ISEMFs on SBS enteroid growth, we examined markers of myofibroblast activation and function in isolated ISEMFs (Figure 8, A and B). SBS ISEMFs exhibited increased *SMA* mRNA expression compared with normal ISEMFs. In contrast, *epimorphin* mRNA expression was decreased in SBS compared with normal patient ISEMFs, consistent with observations in mouse intestinal adaptation after resection (35) and with studies which showed that *epimorphin* deletion in mouse gut results in enhanced crypt cell proliferation (36) and increased enteroid growth in *Epim^–/–^* ISEMF-enteroid cocultures (15). Expression of other mesenchymal markers such as *FOXL1* and *PDGFRα* was unchanged in SBS versus normal ISEMFs.

BMPs are expressed and secreted by ISEMFs; therefore we examined SBS and normal myofibroblast expression of members of the BMP signaling pathway, including *BMP4* and inhibitors *chordin* and *noggin*. In contrast to what we observed in SBS biopsies, which reflects an average increase in *BMP4* expression across all cell types (Figure 5), we observed a significant decrease in *BMP4* mRNA expression in SBS ISEMFs compared with controls (Figure 8C), without change in *noggin* or *chordin* mRNA expression, suggesting ISEMF cell-specific regulation of BMP expression.

**Figure 9:**
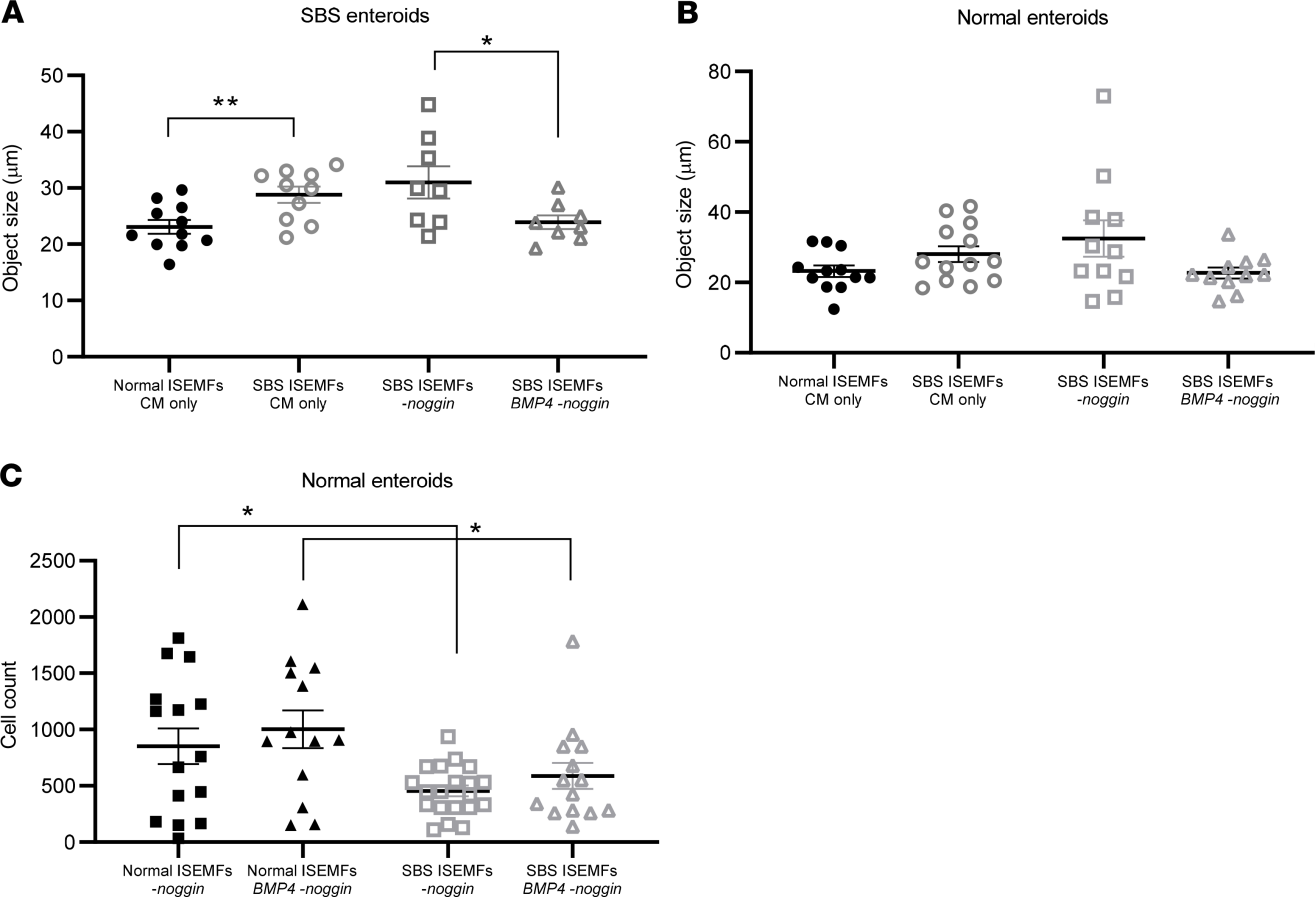
BMP4 reverses the effects of SBS ISEMFs on cocultured SBS enteroid growth. (**A**) SBS enteroids were cocultured with normal human ISEMFs or with SBS ISEMFs in enteroid growth conditioned media (CM), in CM without noggin (-noggin), or in CM without noggin but with BMP4 (BMP4 25 ng/mL, BMP4 -noggin). BMP4 reversed the increase in enteroid size induced by coculture with SBS ISEMFs. **P = 0.0064; *P = 0.04. (**B**) Normal enteroids were cocultured with normal or SBS ISEMFs in CM, in CM without noggin, or in CM without noggin but with BMP4. No change in enteroid size was noted. (**C**) Normal enteroids were cocultured with normal ISEMFs without noggin, with normal ISEMFs without noggin but with BMP4, with SBS ISEMFs without noggin or with SBS ISEMFs without noggin plus BMP4, and cell count was measured. A significant decrease in enteroid cell count was observed in cocultures with SBS ISEMFs. *P = 0.01 normal enteroids with normal ISEMFs versus normal enteroids with SBS ISEMFs; with BMP4; this decrease was not reversed by treatment with BMP4. SBS enteroids, *n* = 4 patients; normal enteroids, *n* = 5–7 patients; normal ISEMFs, *n* = 3 patients; SBS ISEMFs, *n* = 4 patients. Data are mean ± SEM. Statistical analysis by Student’s *t* test.

To determine if reduced BMP4 expression in and secretion from SBS ISEMFs could explain the growth-inducing effect of these cells on SBS enteroids, we cocultured SBS enteroids with SBS ISEMFs, without the BMP inhibitor noggin and with recombinant BMP4 (25 ng/mL; Figure 9) or vehicle. Addition of BMP4 reversed the SBS ISEMF-induced increase in enteroid size (Figure 9A) but had no effect on the size of normal enteroids when cocultured with normal or SBS ISEMFs (Figure 9B). Thus it appears that BMP signaling is inhibited in SBS ISEMF–SBS enteroid cocultures, resulting in increased growth. In contrast, the reduction in the number of stem cell–derived normal enteroids when cocultured with SBS versus normal ISEMFs (Figure 7B) was not reversed by addition of BMP4 (Figure 9C). Thus reduced BMP signaling resulted in an increase in SBS but not normal enteroid growth, but this signaling pathway does not direct the reduction in enteroid number, suggesting regulation of this response by other factors/pathways.

## Discussion

Here we report that massive intestinal resection regulates stem and stromal niche cell populations in the residual gut in humans with SBS. We have analyzed small bowel biopsies as well as in vitro enteroid and stromal ISEMF cell culture models to begin to elucidate underlying mechanisms for the human adaptive response and to identify potential novel targets for therapy. Our study is unique in that we have obtained mucosal small bowel biopsies from a large cohort of patients with SBS, a critically important step forward because this is a clinically heterogeneous population (37). Prior studies (5, 7–11) of the cellular and morphometric features of the human gut adaptive response have been limited by small sample size.

We found that the adaptive SBS intestine has an expanded stem cell population, as shown by stem cell–initiated enteroid formation assays (Figure 3) and by increased expression of *LGR5* mRNA (Figure 1 and Figure 2), the intestinal stem cell marker of the rapidly cycling crypt base stem cell that is responsible for daily renewal of the small bowel epithelium (38). Our analysis of the SBS mesenchyme shows a selective expansion of a subset of stromal cells with putative roles in regulating the stem cell niche. Our data indicate that there is a specific stromal cell adaptive response rather than just a general increase in all stromal cells because we showed that cells expressing CD34/GP38 (Figure 6), which have niche activity in mice (22, 24), were not expanded in intestinal biopsies from human subjects with SBS. We further show that ISEMFs induce growth of cocultured stem cell–initiated enteroids via reduced BMP signaling but only in the setting of SBS.

We found that slower cycling “reserve” +4 stem cell populations marked by SOX9 (28) and BMI1 (39) are not expanded in SBS intestine, findings that contrast with the expansion of LGR5^+^ cells. The biopsies collected in this study were from patients who had SBS for a duration of 6 months up to more than 10 years. It is possible that the reserve stem cell population responds to resection at earlier times after surgery, e.g., in the first few months postresection. These biopsies are more difficult to obtain given the severity of illness early after resection but are required to specify which stem cell population(s) are first mobilized following extensive resection.

Although *LGR5* mRNA expression was significantly increased in small bowel biopsies from SBS versus control patients and stem cell–initiated enteroid formation was greater in SBS patients compared with controls (Figure 1, Figure 2, and Figure 3), we observed patient-to-patient variability for all the analyses. Due to the limited number of samples, we were unable to subdivide our groups to analyze adaptive gene expression in biopsies from patients who had a colon in continuity versus those with ileostomy or jejunostomy, from patients who were on oral versus parenteral feeding, and so on. We also pooled duodenal, jejunal, and ileal biopsies from SBS patients and normal subjects, while recognizing that geographic differentiation could affect the results. However, we focused on analyzing expression of genes that have similar, general functions throughout the gastrointestinal (GI) tract (proliferation, stem cell function, etc.). Also, we performed outlier analyses of all the biopsy-derived mRNA expression data, followed by additional subgroup analysis to see if the outliers had a unique clinical phenotype. Outlier analysis did not reveal a correlation with age, sex, presence or absence of colon, location of biopsy (duodenum, jejunum, or ileum), or oral versus parenteral feeding (i.e., on vs. off TPN). This variability also did not appear to result from differences in the size or mucosal/submucosal depth of the biopsies because there was no consistent pattern of very high or very low expression levels of multiple genes in an individual patient. Similarly, increased expression of epithelial and stromal marker genes in SBS was not simply due to adaptive crypt and villus hyperplasia because multiple markers of the proliferative, differentiating, and stromal epithelium showed no difference in expression levels in SBS versus normal gut (e.g., *SOX9*, *GP38*, *gremlin*, *β-CATENIN*). However, firm conclusions about whether there is a significant association with specific demographic features or with disease process (e.g., Crohn’s disease, ischemia, adhesions) will require analysis of larger numbers of samples per group.

SBS-induced stem cell expansion and increased stem cell function measured by assays of stem cell–derived enteroid initiation (Figure 3) were preserved in these cells following several passages and in assays which used stem cells that had been passaged and were frozen for storage. This phenotypic preservation with passaging and after removal of both luminal and submucosal signals suggests a cell-autonomous mechanism, and specifically, epigenetic modulation has been shown to be responsible for preservation of other phenotypic changes. For example, others have shown that regional small bowel epithelial identity along the horizontal axis (e.g., from duodenum to ileum) is recapitulated in enteroids due to preservation of intrinsic DNA methylation patterns with passaging and in long-term culture (40). DNA methylation profiles were preserved independent of the cellular environment in adult and pediatric organoids, but interestingly fetal organoids showed in vitro maturation, with changes in DNA methylation in culture. Also, disease-specific changes in methylation were identified in organoids derived from a patient with gastric heterotopia, which were also preserved in passaging. Similarly, changes in Crohn’s disease and ulcerative colitis patient enteroid gene expression patterns compared with normal enteroids are also preserved after passaging, likely due to retained epigenetic changes (41, 42). Similar epigenetic mechanisms are likely to be operative in the setting of SBS.

BMP signaling activity is regulated along the crypt/villus axis. BMP inhibitors are produced by pericryptal stromal cells in humans (33). Inhibition of BMP signaling activity is highest at the crypt base where WNT signaling activity is highest, promoting stem cell expansion. BMP4 is expressed in mesenchymal cells adjacent to intestinal stem cells, and noggin is expressed in submucosal cells adjacent to the crypt bottom in mice (43). Stem cell dependence on BMP inhibition is well established, as illustrated by the requirement for exogenous noggin for successful growth of stem cell–derived enteroid cultures in vitro (44). BMP signaling activity increases above the crypt base, from the midcrypt and higher along the crypt/villus axis, to inhibit crypt cell proliferation in the transit amplifying zone and promote epithelial differentiation, counterbalancing WNT signaling activity (30, 45). Mesenchymal BMP expression and BMP signaling activity increase from midcrypt onto the villus (30, 45). BMPs are expressed by mesenchymal cells including ISEMFs (Figure 8 and refs. 16, 34) and Foxl1^+^ telocytes (in mice) (19) and by stromal cells in human colon (BMPs 2, 5, and 7) that have yet to be fully characterized (13). The major small intestinal BMPs are BMP2, BMP4, and BMP7 (45). In mice, BMP4 and BMP inhibitors are expressed by mesenchymal cells whereas BMP2 is expressed by the epithelium (33). In humans, single-cell RNA-Seq shows that *BMP2* and *BMP5* are expressed in a unique subset of colon stromal cells (13); BMP2 and 4 are expressed in human small bowel in epithelium and in stroma, respectively (The Human Protein Atlas, https://www.proteinatlas.org/search/bone+morphogenetic+protein), but cell-specific expression patterns of other BMPS and inhibitors in human small intestine have not yet been elucidated.

Our analysis of SBS versus normal patient biopsies shows an increase in BMP4 mRNA and increased p-SMAD1,5,8, consistent with increased BMP4 signaling overall, which is active on the villus and is required for epithelial differentiation. Yet, our analysis of crypt-derived enteroid-specific interactions with ISEMFs suggests a local, crypt-specific decrease in BMP signaling (Figure 8 and Figure 9). This is consistent with BMP signaling inhibition at the crypt base. We observed that ISEMFs from SBS patients have reduced *BMP4* mRNA expression compared with normal ISEMFs (Figure 8), and incubation of ISEMF-SBS enteroid cocultures with exogenous BMP4 reverses the SBS ISEMF-induced increase in enteroid surface area (Figure 9). Thus the SBS ISEMF stromal cell population responds to resection locally at the crypt, by inhibiting BMP expression, favoring increased crypt cell proliferation.

Cocultures of SBS ISEMFs versus normal ISEMFs with SBS stem cell–derived enteroids induced growth (increased surface area) of cocultured enteroids but not stem cell expansion; the number of stem cell–initiated enteroids in SBS-ISEMF-SBS enteroid cocultures compared with normal ISEMF-SBS enteroid cocultures was not significantly decreased (Figure 7). Also, comparison of normal enteroid–SBS ISEMF cocultures with normal enteroid–normal ISEMF cocultures revealed a significant decrease in the number of stem cell–initiated enteroids but no change in surface area/growth. These results suggest that SBS ISEMFs expand the proliferative transit amplifying cell program, resulting in increased enteroid growth in coculture, but do not have direct effects on the stem cell population. Thus, other stromal cell populations (e.g., FOXL1 telocytes, other PDGFRα^+^ cells; refs. 13, 19, 20, 27) or stem cell/epithelial autonomous mechanisms may regulate the observed expansion in SBS (Figure 3). The observation that coculture of SBS ISEMFs increases growth of SBS stem cell–derived enteroids but not normal enteroids (Figure 7 and Figure 9) suggests resection-induced changes in cell surface growth factor or growth inhibitor receptor expression that are specific to SBS enteroids. Further studies are required to elucidate these mechanisms.

The role of WNT signaling activity in establishing and maintaining stem cell expansion and amplifying population crypt cell proliferation in the human adaptive intestine has not been previously studied to our knowledge. Limited experiments in rodent adaptation following resection show that *Apc^min/+^* mice have an increased crypt cell proliferative response early after resection (e.g., 72 hours after resection; ref. 46), yet Wnt signaling does not play a role in maintaining this morphometric response (47). Others have shown that Wnt signaling is inactive in the mouse gut transit amplifying cell population, although EGFR/RAS/MAPK signaling enhances proliferation of this cell population (27, 48). We found that EGFR expression was significantly increased in SBS biopsies, consistent with a role for this signaling pathway in human SBS. In our present observations of adaptive intestine that has reached a “steady state” following resection, we observed an increase in WNT ligand and agonist expression (*R-spondin 1, R-spondin 2*, and *WNT5a*) without concomitant increased WNT signaling activity (Figure 4); in fact *AXIN2* mRNA expression showed a trend to be decreased (*P* = 0.054), suggesting WNT signaling inhibition. These data suggest that the mesenchyme responds to the loss of small bowel surface area by a compensatory increase in WNT and R-spondin ligand expression but that other counterregulatory mechanisms are activated to inhibit WNT signaling, possibly to protect from chronic WNT stimulation that could result in tumor formation/carcinogenesis. BMP signaling is likely to be one of these counterregulatory mechanisms (43). Our results do not exclude the possibility that WNT-driven expansion of LGR5^+^ stem cells occurred at earlier times postresection, since our patients have had SBS for 6 months to more than 10 years.

The effect of myofibroblasts on cocultured-enteroid growth suggests that examination of other niche cell populations to elucidate effects on stem cell proliferation and expansion could provide novel therapeutic targets for SBS. For example, FOXL1^+^ cells have been proposed to be critically important niche cells for maintaining a normal crypt; however, these cells are relatively rare, making isolation from human intestine challenging. The current findings point to an emerging role of BMP signaling, but clearly other signals are yet to be identified in the adaptive response.

## Methods

### Human studies.

All human studies were performed following protocols approved by the Washington University School of Medicine’s Institutional Review Board, as per IRB 201504100. All studies were conducted according to Declaration of Helsinki principles. Informed consent was obtained for all studies.

### Patient population.

Patients with SBS (*n* = 17; Table 1 and Supplemental Table 1) were recruited from the Gastroenterology Clinic at Washington University in St. Louis School of Medicine or from Barnes-Jewish Hospital in St. Louis. SBS was defined as a residual small bowel length of no more than 200 cm, with or without part or all of the colon in continuity. These included patients with jejunoileal anastomosis and ileostomy, jejunostomy, ileo-colonic anastomosis, or jejunocolonic anastomosis. As per standard practice, all patients were permitted to ingested oral enteral diets even if receiving intravenous (parenteral) nutrition support.

Control subjects (*n* = 27) were recruited from the Center for Advanced Medicine Endoscopy Center at Washington University in St. Louis School of Medicine/Barnes-Jewish Hospital or from the general surgical service. Endoscopy control patients included those who presented for routine colon cancer screening or for routine upper endoscopy for evaluation of esophageal or gastric symptoms but were anticipated to have normal small intestine, which was confirmed during endoscopy. During colonoscopy, the normal terminal ileum was intubated, and a biopsy was taken. Normal jejunum was obtained from patients undergoing intestinal fistula repair; biopsies were taken from normal adjacent mucosa from surgical intestinal specimens removed for fistula repair.

### Human intestinal enteroid isolation and passaging.

Duodenal, ileal, and jejunal biopsy specimens were obtained from healthy patients during routine endoscopy for colon cancer screening, from upper endoscopy for unrelated upper GI symptoms, or from adjacent normal small bowel obtained during fistula repair surgery as detailed above. SBS patient biopsies were obtained during routine clinically indicated endoscopy or from surgical resections performed at Barnes-Jewish Hospital at the Washington University School of Medicine in St. Louis. Patients ingested nothing by mouth for at least 6 hours prior to endoscopy and overnight prior to surgery. Small intestinal enteroids were isolated from biopsy samples as previously described (49). Biopsies were washed with Basic Crypt Media (BCM), consisting of advanced DMEM/F12 with HEPES 10 mM (MilliporeSigma D6421), supplemented with 100 U/mL penicillin/streptomycin (MilliporeSigma), 2 mM Glutamax (Gibco, Thermo Fisher Scientific), and 10% FBS (Gibco, Thermo Fisher Scientific), then cut into small pieces and treated with collagenase type I (Invitrogen, Thermo Fisher Scientific, 17100-017) for 10 minutes at 37°C. Crypts were collected and resuspended in 10 mL BCM, filtered through a 70 μm strainer (MidSci), and centrifuged at 300*g* for 5 minutes. Enteroids were suspended in Matrigel (Corning, 3562237) and plated on 48-well plates. After allowing the crypt-Matrigel suspension to solidify for 10–15 minutes at 37°C, cultures were treated with CM produced from ATCC cell line L-WRN (CRL-3276), which contains Wnt3a (W), R-spondin (R), and Noggin (N), supplemented with 10 μM Y-27632 (p160 ROCK inhibitor, MilliporeSigma, Y0503-1MG, dihydrochloride) and 10 μM SB-431542 (TGF-β receptor antagonist, MilliporeSigma, SB-431542 hydrate). Culture medium was changed every 3 days. Enteroids were passaged to expand stem cells on day 7 after plating by gentle enzymatic digestion in 0.25% Trypsin/EDTA (Gibco, Thermo Fisher Scientific) for 5 minutes at 37°C, replated in Matrigel (50 crypts per 15 μL of Matrigel) into a 48-well plate, and cultured in CM without the addition of 10 μM Y-27632. After up to 7 days in culture, stem cells were frozen for storage.

### Stem cell enteroid initiation assays.

Stem cells were thawed and cultured in Matrigel in CM supplemented with Y-27632 and SB 431542, grown for 7 days, and harvested from Matrigel by enzymatic digestion with 0.25% Trypsin/EDTA (Gibco, Thermo Fisher Scientific) and with pipetting up and down 20–40 times. Live cells were analyzed by staining with 0.4% trypan blue dye (Bio-Rad) and quantified by TC20 Automated Cell counter (Bio-Rad), then replated in Matrigel (50 cells per 15 μL of Matrigel) and grown in CM. Enteroids were imaged with a Zeiss Axiophot microscope with Apotome 2 attachment (Carl Zeiss Inc.) on days 3 and 7 after plating.

### Stem cell initiation assays and quantitation of enteroid growth.

Stem cell–initiated enteroid numbers and size were quantified at 3 and 7 days after plating using Cytation 3 plate reader software (BioTek Gen 3.0). The images were analyzed to quantify total number of viable enteroids per well. The total and average cross-sectional area in the optical level of focus was measured. Enteroid viability was defined by the presence of visually sharp borders along their basolateral (antiluminal) side.

### Small bowel biopsies and ISEMF culture and isolation.

ISEMFs were isolated from duodenal, jejunal, and ileal biopsies, obtained either from healthy patients during routine endoscopy for colon cancer screening or from adjacent normal small bowel obtained during fistula repair surgery and from SBS patients during routine clinically indicated endoscopy or from surgical resections performed at Barnes-Jewish Hospital at the Washington University School of Medicine in St. Louis. Small intestinal biopsies were minced and incubated with 300 U/mL collagenase type I and 0.1 mg/mL dispase (Gibco, Thermo Fisher Scientific) for 2 hours at 37°C; washed with ISEMF culture medium (RPMI 1640 medium from Gibco, Thermo Fisher Scientific) supplemented with 10% FBS, antimycotic solution (Corning), and 10 μg/mL gentamicin (Gibco, Thermo Fisher Scientific); and plated in 6-well plates at 37°C. After ISEMFs attached and formed colonies, they were subsequently passaged, expanded, and plated in standard myofibroblast cell culture medium. This medium consisted of DMEM/Glutamax (Invitrogen, Thermo Fisher Scientific) with 10% FBS (Invitrogen, Thermo Fisher Scientific) and penicillin/streptomycin (MilliporeSigma). The identity and purity of isolated ISEMFs were determined by immunofluorescence to detect expression of α-SMA and vimentin and confirm absence or weak positivity of desmin expression (50). ISEMFs were imaged using a Zeiss Axiovert microscope with Apotome 2 optical sectioning apparatus (Carl Zeiss Inc.).

### Enteroid-myofibroblast cocultures.

ISEMFs from control or SBS patient biopsies were plated into 48-well plates at a density of 1 × 10^4^ cells/cm^2^. Stem cells derived from crypts as above (see *Human intestinal enteroid isolation and passaging*) were frozen for storage and then thawed and cultured in Matrigel in CM supplemented with Y-27632 and SB 431542, grown for 3 days, and harvested from Matrigel by enzymatic digestion with 0.25% Trypsin/EDTA (Gibco, Thermo Fisher Scientific) and with pipetting up and down 20–40 times. Cells were replated in Matrigel (50 live cells per well) onto a 48-well plate, either directly into the well as a monoculture or on top of a preconfluent (~70%) monolayer of ISEMFs cultured in CM (containing Wnt3a, R-spondin, noggin, Y-27632, and SB-431542).

### Immunoblot analysis.

Protein was extracted from phenol-ethanol phase after RNA and DNA precipitation as outlined in the TRIzol (Invitrogen, Thermo Fisher Scientific) manufacturer’s manual. Briefly, an excess of 100% ethanol was added to the phenol-ethanol phase. Samples were vortexed and incubated for 10 minutes at room temperature followed by centrifugation at 12,000*g* for 10 minutes at 4°C. Pellets were washed 2 times in 500 μL 95% ethanol and centrifuged at 7600*g* for 5 minutes at 4°C. After removing ethanol, pellets were air-dried for 5 minutes, and 40–100 μL of TGH protein lysis buffer (Roche Life Science) containing 25 mM HEPES (pH 7.4), 10% glycerol, 1% Triton X-100, and 5 mM EDTA supplemented with complete protease inhibitor cocktail (Thermo Fisher Scientific) was added to samples. Samples were concentrated using the Protein- Concentrate (Micro) kit (catalog 2100, MilliporeSigma). Protein content was quantified using Pierce BCA Protein Assay Kit (Thermo Fisher Scientific), and equal amounts of protein were loaded on NuPAGE 4%–12% polyacrylamide gradient gel (Life Technologies, Thermo Fisher Scientific) under reducing conditions and transferred to nitrocellulose membranes by iBLOT 2 dry blotting system (Life Technologies, Thermo Fisher Scientific). Membranes were blocked and probed with antibodies directed against rabbit anti–p-Smad1/Smad5/Smad8 (Ser463/465) (1:200 MilliporeSigma, Ab3848-I). Mouse anti–β-actin (C4) (1:1000 Santa Cruz Biotechnology, A1713) was used as a loading control. The optical density of the specific proteins was quantified by using LI-COR imaging system (Odyssey CLx infrared imaging system, LI-COR Biosciences).

### Immunofluorescence analysis of ISEMFs.

ISEMFs were cultured on glass slides, fixed in formalin, and processed in situ for immunofluorescence analysis. Staining was performed to identify α-SMA (Abcam 5694, rabbit antibody, 1:200), vimentin (Abcam 8978, mouse antibody, 1:50), and desmin (Abcam 15200, rabbit antibody, 1:50). Anti-rabbit FITC (catalog number 711-095-152), anti-mouse FITC (catalog number 715-096-150), and anti-rabbit Cy3 (catalog number 111-165-144) was each used at 1:200 dilution (Jackson ImmunoResearch Laboratories). Nuclei were counterstained with VECTASHIELD with DAPI (VECTOR Laboratories). Cells were visualized using Nuance microscopy (Nuance Multispectral Imaging System).

### Immunohistochemical analyses.

Biopsies were fixed in formalin, dehydrated in 70% ethanol, and embedded in paraffin. Sections were cut (5 μm) and rehydrated. Heat-induced epitope retrieval (Reveal Decloaker 10x, Biocare Medical) was performed for 20 minutes, followed by 0.3% peroxidase ((HX0635-3 MilliporeSigma), and blocked in blocking buffer (MilliporeSigma) for 1 hour at room temperature. Specimens were incubated at 4°C overnight with mouse anti-Ki67 (1:400, ab156956, Abcam) or mouse anti-PDGFRα (16AI; 1:100, ab96569, Abcam), then washed, incubated for 30 minutes in a detection reagent (HRP-Polymer, Biocare Medical), and developed with DAB (Betazid DAB, Biocare Medical). Sections were counterstained with hematoxylin and visualized and photographed using an Olympus BX43 microscope (Olympus Life Science).

### PDGFRα quantification.

The number of PDGFRα^+^ cells that were subjacent to the villi or to the crypts was quantified. To correct for differences in villus length and crypt depth, the number of positive cells was counted in a rectangular box of fixed area (230 μm × 165 μm), which was overlaid onto photomicrographs of the villi, and crypts of each biopsy were examined. Only intact villi and crypts on each histologic section were analyzed. *P = 0.031 by Student’s 2-tailed *t* test.

### In situ hybridization analyses and quantitation.

A target-specific oligonucleotide (ZZ) probe for Lgr5 was designed (Hs-LGR5-C3; 311021-C3; RNAscope, Advanced Cell Diagnostics, Bio-Techne). Multiplexed Fluorescent Reagent Kit v2-Hs (323135, Advanced Cell Diagnostics, Bio-Techne) was used as per the manufacturer’s protocol. Formalin-fixed, paraffin-embedded tissue blocks were sectioned at 4 μm, then mounted on slides (ColorView Adhesion Slides, StatLab). Slides were pretreated with sequential deparaffinization, hydrogen peroxide, target retrieval, and protease plus digestion, followed by Lgr5 probe hybridization, signal amplification, and detection. Probe hybridization was achieved by incubation of mRNA target probes for 2 hours at 40°C using a HybEZ oven (Advanced Cell Diagnostics, Bio-Techne). The signal was amplified by subsequent incubation of Amp-1, Amp-2, Amp-3, HRP Signal and Opal Dye (Akoya Biosciences)/tyramide signal amplification, 2 drops each for 30, 30, 15, and 30 minutes, respectively, at 40°C using the HybEZ oven. Nucleic acids were stained using DAPI (Advanced Cell Diagnostics, Bio-Techne), and coverslips were mounted with ProLong Gold Antifade Mountant (Thermo Fisher Scientific). The 3-plex negative control probe (Advanced Cell Diagnostics, Bio-Techne, 320871) was applied to separate histologic sections processed in the same manner as those that received the *Lgr5* probe. Slides were visualized under fluorescence microscopy (Zeiss Axiovert microscope with Apotome 2 optical sectioning apparatus by Carl Zeiss Inc.). To analyze the number of Hs-LGR5 molecules per cell in each SBS and normal small bowel biopsy, Lgr5^+^ signals were quantified in each positive cell using ImageJ.

### qRT-PCR.

RNA was isolated from small bowel biopsies using TRIzol (Invitrogen, Thermo Fisher Scientific) reagent according to the manufacturer’s instructions and extracted from ISEMFs using Nucleospin RNA kit (Invitrogen, Thermo Fisher Scientific). Quantification of RNA was performed using 260/280 nm by NanoDrop 2000 (Thermo Fisher Scientific). Complementary DNA was generated from 1 μg/μL of RNA with SuperScript II RT cDNA synthesis kit (Invitrogen, Thermo Fisher Scientific), which was then amplified by PCR 2720 Thermal Cycler (Applied Biosystems, Thermo Fisher Scientific). Gene expression was determined by StepOnePlus system (Applied Biosystems, Thermo Fisher Scientific) using Fast SYBR Green Master Mix (Applied Biosystems, Thermo Fisher Scientific) for all PCRs. Primers used are listed in Table 2. Cycle threshold values were normalized to 18s mRNA levels. The fold induction was determined by the ΔΔCt method (51).

### BMP4 effects on enteroids and enteroid-ISEMF cocultures.

Stem cells were removed from frozen storage and incubated in enteroid CM with Wnt3a, R-spondin, SB-431542, and Y-27632, with or without noggin. Media were changed 3 days postplating. Imaging and quantitation of surface area and cell count were performed at 3 days postplating. ISEMF-enteroid cocultures were incubated in CM as above, with or without Bmp4 (25 ng/mL; recombinant human BMP4, 314-BP: R&D Systems, Bio-Techne), but without noggin. Cocultured enteroids were analyzed for size (surface area) and cell count at 3 days postplating.

### Statistics.

Data comparing mRNA expression and enteroid size and cell count from normal patient and SBS biopsies or from ISEMFs and enteroids were calculated as the mean ± SEM. Statistical significance was analyzed by 2-tailed Student’s *t* test (GraphPad, Prism 8) comparing normal with SBS samples. *P* values less than or equal to 0.05 were considered significant, and *P* values are reported in each figure legend.

Outlier values were determined using the rule that defines an outlier as a value larger than 1.5 times the interquartile range from the 75th percentile or smaller than 1.5 times the interquartile range from the 25th percentile. The subject with at least 1 outlier in the gene group was defined as an outlier. The association between outlier distribution and demographic variables was examined using 2-sample 2-tailed Student’s *t* test for continuous demographic variables and χ^2^ test for categorical demographic variables.

### Study approval.

All human studies reported in this manuscript were approved by the Institutional Review Board of Washington University in St. Louis, as indicated in human studies protocol IRB 201504100. Written informed consent was received from participants prior to inclusion in the study.

## Author contributions

VAG designed research studies, performed experiments, analyzed data, and contributed to writing manuscript. EAS designed research studies, performed experiments, and analyzed data. MUL performed experiments and analyzed data. AS performed experiments and analyzed data. RD performed experiments and analyzed data. MG performed experiments and analyzed data. DMA provided reagents and contributed to designing research studies. MAC provided reagents. GB provided reagents. OI provided reagents. JK provided reagents. WJS provided reagents. NOD designed experiments and wrote the manuscript. MSL designed experiments and wrote the manuscript. DCR designed experiments, collected data, analyzed data, and wrote the manuscript.

## Supplementary Material

supplemental data

## Figures and Tables

**Table 2:**
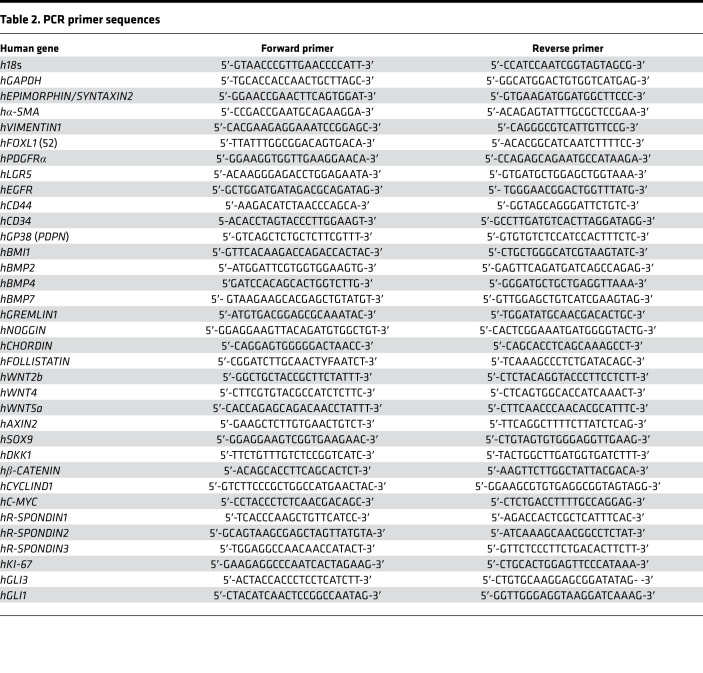
PCR primer sequences

**Table 1:**
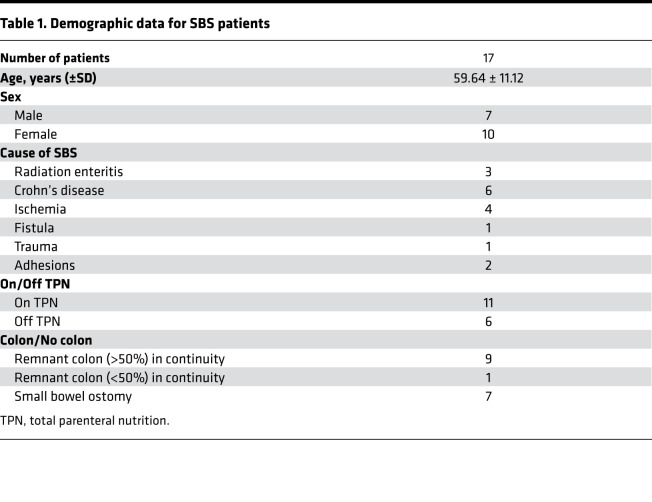
Demographic data for SBS patients

## Version 1 11/03/2020

In-Press Preview

## Version 2 12/03/2020

Electronic publication
