# Supplementary material for: Stem cell and niche regulation in human short bowel syndrome
Source: JCI Insight. 2020 Dec 3;5(23):e137905. doi: 10.1172/jci.insight.137905 (PMC7714413; doi:10.1172/jci.insight.137905)

Supplemental Table 1: Demographics of short bowel syndrome patients

Gender	***Age (years)***	***Race***	***Cause of SBS***	***On/Off TPN***	***Colon present (yes/no)***	***SBS duration***
F	76	White	Radiation enteritis	On TPN	Yes (<50%)	30y
F	44	White	Crohn's	On TPN	No	6m
F	66	White	Crohn's	Off TPN	No	11m
F	64	White	Perforation	Off TPN	No	12m
M	69	White	Ischemia	On TPN	Yes	10y
F	52	White	Fistula	On TPN	Yes	12m
M	72	White	Crohn's	On TPN	No	21m
F	71	White	Radiation enteritis	On TPN	Yes	4y
M	37	White	Ischemia	Off TPN	Yes	4y
M	65	White	Trauma	On TPN	Yes	2y
F	60	White	Adhesions	On TPN	Yes	10y
F	47	White	Ischemia	On TPN	Yes	10m
F	61	White	Adhesions	On TPN	Yes	20m
M	68	White	Crohn's	Off TPN	No	4y
M	53	White	Crohn's	Off TPN	No	6y
M	47	White	Crohn's	Off TPN	No	10y
F	62	White	Radiation enteritis	On TPN	Yes	1y

N=17 patients; y=years, m=months

Supplemental Figure 1

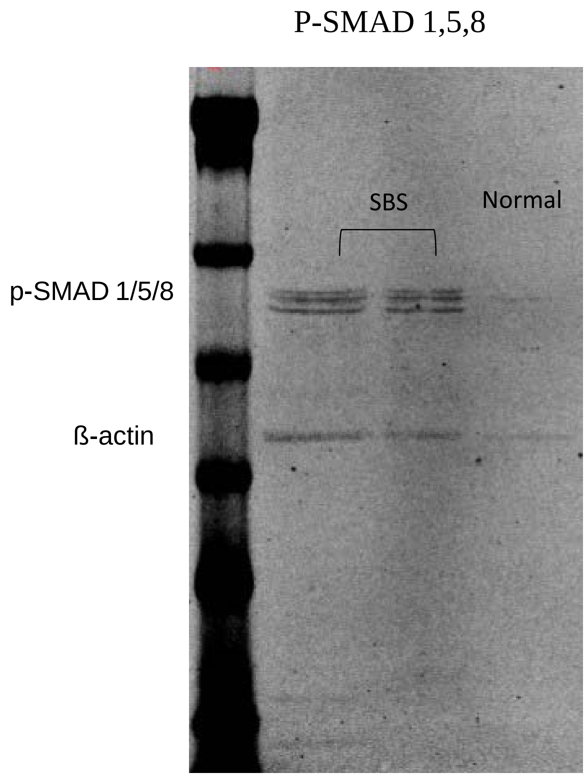

Supplement: supplemental data [file jciinsight-5-137905-s020.pdf]
